# Using Experience Based Co‐Design to Develop a Novel Psychological Intervention With People With Intellectual Disabilities and Stakeholders

**DOI:** 10.1111/jar.70022

**Published:** 2025-02-13

**Authors:** Olivia Hewitt, Peter E. Langdon, Michael Larkin

**Affiliations:** ^1^ Intellectual Disabilities Research Institute (IDRIS) University of Birmingham Birmingham UK; ^2^ Erlegh House Berkshire Healthcare NHS Foundation Trust Reading UK; ^3^ Institute for Health and Neurodevelopment, Department of Psychology Aston University Birmingham UK

**Keywords:** anxiety, co‐design, experience based co‐design, intervention, mental health

## Abstract

**Background:**

Psychological interventions need to be adapted for use with people with intellectual disabilities to ensure they are engaging, accessible and effective. Co‐design allows the experiences of service users and stakeholders to actively shape and develop interventions, to ensure their accessibility.

**Method:**

An adapted model of Experience Based Co‐Design (EBCD) was used to co‐develop a novel, mental imagery‐based psychological intervention for people with mild to moderate intellectual disabilities and anxiety. Involvement in EBCD was evaluated for people with intellectual disabilities and stakeholders using both quantitative and qualitative methods.

**Results:**

Numerous concrete and specific intervention adaptations arose and were implemented. Our findings indicated that all participants were able to engage fully with EBCD, and that participants found the process a positive experience.

**Conclusions:**

EBCD has likely resulted in a more accessible and engaging intervention which can be now tested within a larger study.

## 1 Introduction

People with intellectual disabilities face numerous health inequalities (Emerson 2021) and rates of mental health conditions are several times higher than in the general population (e.g., Hughes‐McCormack et al. 2017; Buckles et al. 2013). Higher rates of anxiety disorders are seen across people with intellectual disabilities of mixed aetiology, with even higher rates in syndromic intellectual disabilities (Edwards et al. 2022).

Despite this, developing accessible, effective psychological interventions for this population has been challenging, with careful adaptations needed to ensure people can access treatments. Most psychological interventions, such as cognitive behavioural therapy (CBT), require adaptation for people with intellectual disabilities, and there is evidence to support their use. For example, systematic reviews and meta‐analyses indicate that CBT is likely an effective intervention for anger and depression in people with intellectual disabilities (e.g., Coooper et al. 2016; Vereenooghe and Langdon 2013; Tapp et al. 2023), but the literature is fraught with methodological bias limiting the certainty of such a conclusion. There are few controlled studies evaluating CBT for anxiety in people with intellectual disabilities with much of this literature consisting of small studies with methodological weaknesses (Unwin et al. 2016; Dagnan et al. 2018).

CBT approaches for people with intellectual disabilities are often adapted to focus on behavioural, rather than cognitive strategies to reduce the reliance upon ‘talking’ therapy components. The cognitive limitations of people with intellectual disabilities may make some cognitive techniques (e.g., identifying and altering core beliefs) less accessible, as they require sophisticated metacognitive and verbal skills (e.g., Tsimopoulou et al. 2018). Interventions for mood disorders (such as anxiety) may also focus on behaviour management strategies rather than adapting internal cognitions (Acton et al. 2023), as changes in behaviour can be more easily identified by others.

Traditional CBT approaches have focused on verbal rather than visual cognitions (mental imagery). Mental images are internal mental processes whereby perceptual information is accessed from memory, rather than directly from external stimuli. They can occur in one or more sensory modalities and have been described as ‘seeing with the mind's eye’ or ‘hearing with the mind's ear’ and so on (Kosslyn et al. 2006). Distressing or intrusive mental imagery is associated with the development and maintenance of various psychological disorders (Hackmann et al. 2011), and various mental imagery interventions have been developed to treat psychopathology, including among those with limited cognitive skills (e.g., Schwarz et al. 2020). These techniques include imaginal exposure and systematic desensitisation, imagery rescripting, guided imagery, and promoting positive imagery (Holmes and Mathews 2010). While mental imagery interventions form a core part of psychological treatments for disorders (including anxiety disorders), they have not yet been adapted or investigated for use with people with intellectual disabilities.

Co‐design is increasingly being used within mainstream services to develop new interventions and to improve clinical services (e.g., Masterson et al. 2022). It aims to develop a detailed understanding of how an intervention is experienced by service users and stakeholders, thus allowing barriers to engagement, and other issues around acceptability and accessibility to be identified and ameliorated at the design stage (Williamson et al. 2021). However, co‐design is costly (in terms of time and resources) when conducted with people who have or do not have intellectual disabilities (Hewitt, Langdon, Tapp et al. 2023). While co‐production is increasingly being used to develop complex interventions (Smith et al. 2022), only one psychological intervention to date has been co‐produced with people with intellectual disabilities (Acton et al. 2023). Other psychological interventions for anxiety among people with moderate to severe intellectual disabilities have been developed collaboratively with clinicians and carers (Langdon et al. 2024a; Langdon et al. 2024b).

Various models of co‐design have been developed. Within mental health services, Experience Based Co‐Design (EBCD) has been used to co‐develop new services (Hawke et al. 2024), and adapt psychological and behavioural interventions for various physical and mental health conditions including perinatal depression (Pinar et al. 2022) and those surviving critical physical illnesses (da Silva et al. 2024). EBCD has been used to improve services for people with intellectual disabilities (e.g., Dimopoulos‐Bick et al. 2019), and adaptations have been made to reduce the power imbalance, and to accelerate the EBCD process (Heerings et al. 2022).

Recently, Green et al. (2020) suggested EBCD could be employed to design complex research interventions, increasing intervention person‐centredness and successful implementation. Fylan et al. (2021) produced a comprehensive protocol for using EBCD for intervention development and evaluation. Therefore, EBCD was the methodology we chose to co‐design a novel, mental imagery‐based psychological intervention for anxiety in people with mild to moderate intellectual disabilities. In developing this intervention, much of the guidance suggested by Fylan et al. (2021) has been followed but only the intervention design phase is described within this paper. Subsequent phases, including the intervention user testing and refinement phases are currently being conducted alongside people with intellectual disabilities and stakeholders; our findings will be reported in the future. Our focus here is upon describing the use of an adapted version of EBCD to design the intervention for use with people with intellectual disabilities. Additional changes were required when using EBCD to ensure that the process was meaningful and accessible which are described in this paper.

### 1.1 Aims

This paper aims to (a) describe how EBCD was adapted to co‐design an intervention with people with intellectual disabilities and stakeholders, (b) describe the resulting intervention refinements made to meet the needs of people with intellectual disabilities, and (c) understand the experiences of participating in EBCD and consider whether this methodology was acceptable and accessible for all participants.

## 2 Method

### 2.1 Participants

Two groups of participants were recruited: (1) a group of stakeholders, made up of family members, health and social care professionals, service commissioners, advocates and those working directly with people with intellectual disabilities in support roles. Stakeholders included people who had been involved with previous phases of the wider research project, and those who with no previous involvement, and (2) a group of people with mild to moderate intellectual disabilities (some of whom had been involved in previous aspects of the project, and some had had no prior involvement). Both stakeholders and people with intellectual disabilities were recruited through community groups such as self‐advocacy groups, and community projects.

Inclusion criteria for the group of stakeholders were: (a) self‐identifies as someone who is a stakeholder for people with intellectual disabilities and services provided to support them, (b) provides informed consent to participate, and (c) has adequate English language skills to participate in interviews and discussions. Exclusion criterion for the group of stakeholders was being unable to provide informed consent to participate in the study.

Inclusion criteria for the group of people with intellectual disability were: (a) diagnosis of mild or moderate intellectual disability, (b) self‐identifies as someone with a mild or moderate intellectual disability and is recruited through services specifically for people with intellectual disabilities, (c) has adequate English language skills to participate in interviews and discussions, (d) is able to provide informed consent to participate in the research, and (e) is aged 18 years or older.

Exclusion criteria for the group of people with intellectual disability were (a) having a diagnosis of severe or profound intellectual disability, (b) known to have been assessed as not having an intellectual disability or has been refused access to health or social care services for people with an intellectual disability, (c) aged under 18 years, or (d) unable to participate in verbal interviews.

The group of people with intellectual disabilities included those with various additional physical and sensory needs, and some with personal experience of mental health difficulties and receiving psychological interventions. Two potential participants approached the research team via email to request participating as a member of the group of people with intellectual disabilities. The research team clarified the inclusion criteria of having a diagnosed intellectual disability with these individuals, who did not make further contact with the research project.

While participant's level of intellectual disability was not assessed to establish a diagnosis of mild or moderate intellectual disability, only participants who were currently, or had historically received a service from the community learning disability team were included. The team would have previously determined that each participant had a learning disability and therefore it was not necessary for the research team to repeat this process. People with a severe or profound intellectual disability were excluded from the study as they were unable to provide informed consent to participate.

**FIGURE 1:**
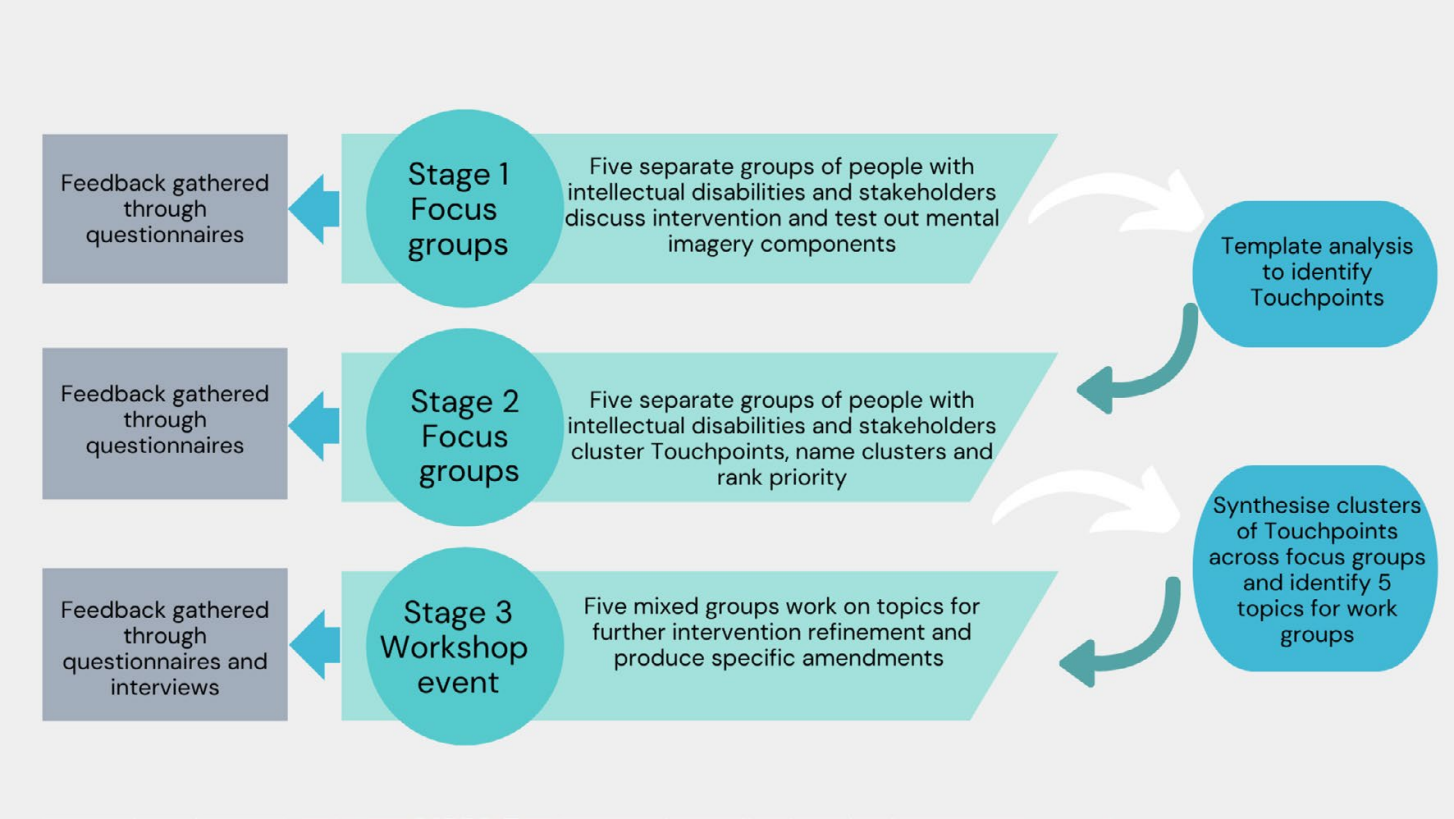
The three stages of the Experience Based Co‐Design process.

Participants could be involved in one or more focus group (Stage 1 and Stage 2) and/or the workshop event (Stage 3) (see Figure 1).

A total of 61 individuals took part across all three stages of the project. All participants in Stage 1 and 2 focus groups were paid £10 to thank them for their time. No payment was made for Stage 3 attendance due to budgetary constraints. All participants in the project provided written, informed consent to participate prior to their inclusion in the project. A favourable ethical opinion for this phase of the project was provided by the Humanities and Social Sciences Research Ethics Committee, University of Warwick (HSSREC 124/20‐21).

Table 1 details demographic information for the participants involved in each stage of the EBCD process.

**TABLE 1:** Demographic information for participants.

	Stage 1 focus group	Stage 1 focus group	Stage 1 focus group	Stage 1 focus group	Stage 1 focus group	Stage 2 focus group	Stage 2 focus group	Stage 2 focus group	Stage 2 focus group	Stage 2 focus group	Stage 3 workshop
Number of people with Intellectual Disability	*N* = 3	N = 3	N/A	N/A	N/A	*N* = 3	*N* = 4	N = 3	N/A	N/A	*N* = 16
Additional diagnoses	Complex PTSD	Epilepsy, anxiety	N/A	N/A	N/A	Complex PTSD	Deaf, epilepsy, anxiety	Anxiety	N/A	N/A	Epilepsy, complex PTSD, anxiety, deaf, cerebral palsy
Age range	43–54	25–48	38–44	N/A	N/A	43–54	25–56	18	N/A	N/A	22–63
Ratio F:M	3F	2F:1M	2F	N/A	N/A	3F	2F:2M	2F:1M	N/A	N/A	F12:M4
Number of stakeholders	N/A	N/A	*N* = 2	*N* = 3	*N* = 4	N/A	N/A	N/A	*N* = 5	*N* = 5	*N* = 32
Role	N/A	N/A	Learning disability nurse, commissioner	Day service manager, family member, clinical psychologist	Clinical psychologist, researcher, family member	N/A	N/A	N/A	Learning disability nurse, commissioner, day service manager, family member	Advocate, researcher, clinical psychologist, family member	Clinical Psychologist, family member, support worker, day services manager, researcher, educational worker
Age range	N/A	N/A	N/A	41–62	24–43	N/A	N/A	N/A	27–62	25–53	Age 20–83
Ratio F:M	N/A	N/A	N/A	2F:1M	4F	N/A	N/A	N/A	4F:1M	4F:1M	27F:5M

### 2.2 Design and Procedure

Before embarking on the process of EBCD, the research team set three parameters to the intervention that was to be developed. This was required to present participants in the EBCD process with materials and examples of potential aspects of the intervention that could be included or adapted. One way we made EBCD accessible for people with intellectual disabilities was to present ideas and examples of potential aspects of the intervention (e.g., different outcome measures, examples of mood recording sheets, examples of mental imagery interventions) that could be included or amended. Asking people to design such materials from scratch or be aware of existing materials that could be included would be too time‐consuming and complicated.

The three parameters were that the intervention should be accessible for people with mild to moderate intellectual disabilities, target symptoms of anxiety, and be based on one or more mental imagery components. People with severe and profound intellectual disabilities are unable to access talking interventions, and therefore we did not require the intervention being developed to be accessible to these groups of people. Additionally, we do not know what experiences of mental imagery people with severe and profound intellectual disabilities can experience, manipulate and report. Anxiety was chosen as the focus of the intervention because it is common in people with intellectual disabilities, and because people with anxiety disorders have more frequent and accessible mental images than in other mood disorders (Ng et al. 2015). A novel aspect of the intervention was that it should be based on mental imagery interventions, as previous work (Hewitt et al. 2022; Hewitt, Langdon, Hales et al. 2023) indicated that these may be effective and accessible methods to shift affect in people with intellectual disabilities and mood disorders. Therefore, examples of three different types of mental imagery interventions were chosen to be presented through the EBCD process. These specific mental imagery interventions were chosen because they have a good evidence base in mainstream populations, have clear mechanisms of change described in the literature and are underpinned by a robust psychological theory, and could be easily adapted to be accessible for people with mild to moderate intellectual disabilities. The interventions were chosen after discussion within the research team and through consulting expert clinicians who deliver mental imagery interventions to other client groups. The mental imagery interventions that were selected were generating positive images (Brougham et al. 2020), the attention training technique (Wells 1990) and changing the properties of an image (based on imagery rescripting exercises; Holmes et al. 2019). The types of mental imagery manipulation required by each mental imagery component were also considered. It was hoped that by including relatively simple mental image exercises such as generating an image (as in generating positive images and the attention training technique), as well as exercises which required several mental imagery processes such as generating, inspecting and transforming an image (such as changing aspects of an image), we would have imagery components that would be accessible to individuals with a range of mental imagery abilities. It was expected that one or more of these mental imagery components would be selected and adapted for inclusion in the intervention through EBCD.

No other parameters were set before the EBCD process, leaving scope for the intervention to be developed in a myriad of creative ways, depending on the input from EBCD participants. Despite the constraints of the three parameters that had been set, participants within the EBCD process continued to provide feedback and reflections on how these limitations may be addressed in future work, which are considered within the discussion section.

There are detailed and well‐established guidance for using EBCD as a quality improvement tool (e.g., through The Point of Care Foundation). As mentioned earlier, Fylan et al. (2021) provide details of how EBCD can be integrated into intervention development and evaluation. Within this project, our priority was for the EBCD process to be accessible and meaningful for people with intellectual disabilities, as well as stakeholders. Therefore, Table 2 details the phases of EBCD as used for intervention development (Fylan et al. 2021) and the adaptations made within this project to ensure accessibility for people with intellectual disabilities. These were based on literature describing how best to adapt psychological interventions for people with intellectual disabilities (Witwer et al. 2022), the clinical experience of the research team in working with this client group, and previous research phases including a systematic review of mental imagery in people with intellectual disabilities (Hewitt, Langdon, Tapp et al. 2023) and a phenomenological study into the experience of mental imagery in people with intellectual disabilities (Hewitt, Langdon, Hales et al. 2023).

**TABLE 2:** Adaptations to intervention development EBCD processes made in this project.

EBCD intervention development stage and purpose (Fylan et al. (2021)	Adaptations made in current project
	Generic adaptations to improve accessibility, such as: Taking extra time to explain the context of the project and the task participants were being asked to complete at each stage of the project, Using visual aids to help explain concepts. Building rapport and allowing extra time to do this. Emphasising that there are no wrong or right answers and that all feedback is welcomed All facilitators had clinical experience of working with people with intellectual disability Ensuring meetings took place in a familiar, convenient and accessible location. Meeting people in a venue they felt comfortable, that is, for one focus group we met at a day centre as this was the most convenient location for everyone.
Stage: Participant training (as an event or distributed in stages) Purpose: To inform and engage participants and investigators with respect to EBCD	Information and training around EBCD were provided to stakeholders and people with intellectual disabilities separately. Smaller amounts of information, targeted to be directly relevant to the current EBCD stage, were provided regularly (every 6–8 weeks) throughout the project. Sessions were provided face‐to‐face for people with intellectual disabilities and online for stakeholders to improve accessibility.
Stage: Exploratory research Purpose: To understand the lived experience of patients, their supporters or carers and providers	A formal qualitative study using Interpretative Phenomenological Analysis (Hewitt, Langdon, Tapp et al. 2023) had previously been conducted into the mental imagery experiences of people with intellectual disabilities. A systematic review of mental imagery in people with intellectual disabilities had been conducted (Hewitt, Langdon, Hales et al. 2023). Results from both of these studies fed into the EBCD project. Stage 1—Separate focus groups were conducted with small groups of people with intellectual disabilities and stakeholders to explore their experiences of undertaking various aspects of the intervention. Focus groups were deliberately kept small (3–4 participants with intellectual disability) to allow plenty of time and space for everyone to participate, and so a higher level of facilitation could be provided if required These were formally analyzed using Template Analysis, synthesised across both groups and touchpoints identified. Touchpoints were refined to reduce duplication, simplify the language used and ensure each touchpoint contained only one concept.
Stage: ‘Trigger film’ production Purpose: Create a ‘trigger film’. This is a short video which summarises key salient moments (touchpoints) from a patient and carer perspective	This was completed after Stages 1 and 2 and before Stage 3. The trigger film consisted solely of people with intellectual disabilities, who interviewed each other about the emotional touchpoints of the project, their experiences of anxiety and the consequences of untreated mental health conditions on their everyday life.
Stage: Patient and carer event. Purpose: First showing of trigger film to patients and carers, discuss priorities for service improvement	Stage 2—A further series of small focus groups were held with people with intellectual disabilities separately to cluster the identified touchpoints into areas for intervention refinement. These areas were then ranked in order of priority for change. Facilitators had flexibility so that if required, they could provide extra support and guidance to the groups of people with intellectual disabilities when they were clustering and prioritising touchpoints, as this was felt to be a challenging task. However, these groups coped very well with the task, and generated their own strategies to help complete the task, such as using a pen to highlight keywords on each touchpoint which helped them to cluster similar ideas.
Stage: Professional event Purpose: Discuss priorities for service improvement with providers	Stage 2—A further series of small focus groups were held with stakeholders separately to cluster the identified touchpoints into areas for intervention refinement. These areas were then ranked in order of priority for change.
Stage: Joint event Purpose: Second showing of trigger film to all stakeholders, discuss priorities for service improvement	Stage 3—The joint workshop event was held at an accessible community venue. This included the first showing of the trigger film. Priorities for intervention refinement had been established in Stage 2 focus groups, to allow people additional time and smaller groups in which to complete this task. Therefore, the areas for intervention refinement had already been identified ahead of the workshop, allowing for careful planning regarding the makeup of each small group. This ensured everyone had the necessary support to be able to engage with the task (e.g., familiar support worker in same group).
Stage: Design groups Purpose: Develop solutions for the identified service improvement priorities	Stage 3—During the workshop mixed groups of participants worked in small groups to develop solutions regarding five different areas for intervention refinement. Extensive feedback on the planning of the workshop was sought from all potential participants to ensure the day was as accessible as possible. We ensured that each small work group had at least two (and usually three or four) people with intellectual disabilities on each table, so no one individual was responsible for providing the lived perspective of having an intellectual disability. People were welcome to bring a supporter and could also specify if they wished to be in the same group as their supporter. Each group was provided with an accessible task (with minimal literacy skills required) that prompted discussion and solution generation for each area of intervention refinement. Alternative formats to written exercises, such as role‐plays, pictures or symbols were used. Similarly, feedback could be provided in a picture/symbol format, and through voice notes or short video clips. Particular attention was paid to the venue, timings, and catering arrangements. Frequent breaks and opportunities for networking and building community were commonly highlighted as important in feedback. Flexibility around timings and tasks was essential, as some groups finished their tasks early, while others struggled to complete their tasks in the allotted time. Additional strategies to help facilitate engagement and recognition of the group included the use of colour coding each group (balloons at the table, all materials produced were colour coded, participants name badges etc.). Each group had two skilled facilitators who had extensive experience of working with people with intellectual disabilities and knowledge of EBCD.
Stage: Celebration event Purpose: Share and further develop service pathways and improvement tools	Additional ideas for intervention refinement were collated following the workshop and changes made to the intervention. These were then shared with stakeholders in a written format and feedback provided. Further changes were subsequently made to the intervention. This allowed more specific feedback around different aspects of the intervention to be gathered from those with the most relevant experience.
Stage: Design for feasibility testing Purpose: Check existing evidence for proposed solutions, map on to chosen theory and create causal model and fully develop prototypes	Feedback was sought from stakeholders and people with intellectual disabilities regarding the additional materials produced to support intervention delivery. This was gathered through individual and small group discussions to improve accessibility (eliminating the need for literacy skills). Other intervention refinements were also shared with stakeholders in a written format, with feedback received and acted on.
Stage: Feasibility/ pilot and main study Purpose: Implement, refine and test solutions in experimental research study	Initial testing of the intervention is currently occurring in NHS settings using single case experimental design with people with mild to moderate intellectual disabilities and anxiety.
Data and analysis from earlier qualitative and/or quantitative exploration in this context and findings from the broader literature are always available)	We found the use of previous qualitative studies and a systematic review, as well as consultation with the broader literature was invaluable in feeding into all stages of this process.

#### 2.2.1 Stage 1

Initially a series of five focus groups were conducted, each lasting around 1 hour. Three focus groups consisted of stakeholders, and two focus groups of people with intellectual disabilities. Each focus group was facilitated by a clinical psychologist with 17 years' experience of working clinically with people with intellectual disabilities. The facilitator was able to use open‐ended questions as well as prompts and examples to ensure that participants understood the information being presented to them. Building a good working relationship with participants ensured they felt able to express uncertainty and to ask questions and for clarification. It was made clear to participants that there were no right or wrong answers and that the facilitator was interested in hearing all of their ideas, and especially any concerns or areas of confusion regarding the intervention. The facilitator made sure to provide feedback and positive reinforcement for all contributions from participants and to encourage discussion between participants.

At the start of the group, the three parameters of the intervention set by the research team were outlined and the rationale for these was provided. Participants were invited to discuss all aspects of the intervention. A specific aim of the focus group was for participants to understand the three mental imagery components of the intervention, and to discuss how these could be presented to patients. The exercises proposed to teach each mental imagery component were explained and tried out in the focus group, and feedback was provided. The first mental imagery component is that of generating positive mental imagery. This is done through one of two exercises adapted for people with intellectual disability and based on Compassion Focused Therapy. To demonstrate these two exercises, the facilitator read each exercise from a script and encouraged the participants to engage with the exercise in the room. The two exercises were ‘Calm Place’ and ‘Kind Helper’ (adapted perfect nurturer exercise; Lee 2005). The second mental imagery component is that of switching attention from an internal image to external stimuli. This was demonstrated though guiding participants through a grounding exercise (in which they were asked to notice five things they could see of a specific colour, and work through the other senses to focus attention in the room). It was also demonstrated through use of multi‐sensory objects such as different essential oils to smell, different sensory objects to touch, and so forth. Finally, participants were guided through an exercise to change the properties of an image. This was demonstrated through viewing a short film clip which demonstrates the technique. Additionally, group discussions concerned the logistics of providing an accessible talking therapy for people with intellectual disabilities, the materials that should be covered within the intervention (e.g., providing psychoeducation around anxiety), and how people could be helped to remember what new skills they had learned in the therapy. These were elicited through the focus group facilitator asking open‐ended questions following on from comments made by participants, and subsequent spontaneous conversations between group participants.

Each focus group was audio recorded, transcribed, and analyzed using Template Analysis (Brooks et al. 2015). In addition to Template Analysis, the transcripts were analyzed to identify ‘touchpoints’. Touchpoints are emotionally charged or key moments and have been defined as any crucial moment that makes a difference (good or bad) to someone's experience of the environment or process (Robert 2013). Initially 67 potential touchpoints were identified. These were refined to remove duplicate ideas, and to simplify the language used. Discussion among the authors and with stakeholders involved in steering the overall project resulted in the touchpoints being reduced to 46 touchpoints.

#### 2.2.2 Stage 2

A second series of five focus groups (three with people with intellectual disabilities and two with stakeholders) were held to consider the identified touchpoints. Each focus group lasted around 1 hour. Each touchpoint was printed onto a card in large type and placed upon a table in front of the participants. Each focus group was asked to cluster together touchpoints into meaningful groups. To facilitate this process for people with intellectual disabilities, these groups chose to use a pen to underline any key words in the touchpoint and this helped them to group together touchpoints that referenced similar concepts. Once the touchpoints were clustered together, the group named each cluster with a title that summarised the concepts it contained. Groups were then ranked each cluster in terms of its priority for action. Generally, groups chose to move these clusters around the table, physically representing their order of importance.

Following these five focus groups, 33 clusters were identified and ranked by the groups. Most groups had clustered their touchpoints in similar ways, meaning there was a large amount of overlap between these 33 clusters. The 33 clusters were synthesised by OH and discussed with ML. This resulted in eight themes of synthesised clusters being identified. The five most prevalent clusters were then taken forward to the workshop (Stage 3) to form the topics for small group work.

#### 2.2.3 Stage 3

A one‐day workshop was held in an accessible community venue. This was attended by 48 participants, including 16 people with intellectual disabilities. The day consisted of viewing a series of short trigger films made by people with intellectual disabilities who were part of the steering group for the overall project. These films focused on their experience of anxiety, the impact anxiety had on their everyday life, and their thoughts about engaging with the mental imagery intervention. Trigger films are used to generate discussion and to support priority setting around intervention adaptation. For example, a person talking about feeling misunderstood and isolated by anxiety may have led to discussion around the importance of psychoeducation within the intervention, whether having a supported attending therapy sessions could reduce isolation, or whether additional strategies such as developing social networks should be included in the intervention.

During the workshop, five groups consisting of people with intellectual disabilities and stakeholders explored topics to further refine the intervention. Each group was given a colour as well as a name to help aid recognition for all group members. Paperwork, badges and balloons at each table were similarly colour coordinated to help participants identify their own table and topic. The five most prevalent themes from Stage 2 were taken forward as the topics for this small group work. Each small group spent time completing a task to help answer or address the outstanding issue that required resolution. A list of the small group topics, their aims, and the activities to address these aims is given in Table 3. Each small group completed a task to resolve the outstanding issue, and discussion among all members so the group helped to explore alternative amendments, before clarifying the groups decision about how best to resolve the issue. Group discussion was facilitated by two group members who had in depth knowledge of the issue being presented, and of working with people with intellectual disability. Their facilitation ensured all group members understood the task and were able to contribute their ideas to the discussion. Following the 2 hour long sessions of group work (before and after lunch), each group completed a decision grid that specified ‘What action we have decided should happen?’, ‘Why did we decide this?, ‘Who will do it?’, ‘By when?’, ‘Any barriers that might get in the way?’, and ‘What will be different when it has been done?’. This helped to detail each recommendation made by the group to further develop the intervention. In addition, each group facilitator fed back the content of the group discussions, to detail what ideas were taken forward by the group as well as those rejected. Each facilitator also captured their own reflections on the process which were discussed by the research team.

**TABLE 3:** Details of small group work completed during the workshop.

Name of workgroup	The role of supporters	Accessibility and engagement	Understanding anxiety	Measuring anxiety	Mental imagery components
Colour	Green	Pink	Orange	Purple	Blue
Aim	How can we help people identify the right supporter? How can we balance confidentiality with sharing information with supporters?	How to make the intervention as interesting and fun as possible? How to make the materials as accessible as possible?	What do people need to know about anxiety before we start the intervention? How should we help them to know about this?	How can we help people report how they are feeling every day?	How to make the mental imagery exercises as accessible as possible? How to help people practice these skills outside sessions?
Task	Watch role plays illustrating examples of good and bad supporters	Add sticky notes to a visual plan of the intervention to identify areas to improve accessibility and engagement	Review different anxiety psychoeducation materials and decide which should be included in the intervention.	Consider different ways of reporting the person's mood on a daily basis.	Go through mental imagery exercises and give feedback on accessibility. Generate ideas to help practice skills between sessions.
Example of decisions made by the group	To add information about what a good and bad supporter looks like to the client workbook. Include information about what to do if unhappy with their supporter. Therapist to go through this information with them at beginning of therapy	Add multi‐sensory items e.g., music, scents to the therapy (if the client enjoys these) to enhance mental imagery and increase engagement. Explore these ideas in session 4 and include in blueprint if helpful.	Provide written information about anxiety at screening	Need flexible options for daily recording. Consider Format of recordings (electronic/paper/app) What support required to set this up?	Record the therapist talking through the calm place/kind helper exercises so the person can use to practice these in between sessions.

#### 2.2.4 Capturing Feedback on the EBCD Process

To understand the experiences of those involved with the EBCD process, questionnaires and semi‐structured interviews were employed. Quantitative questionnaires were administered to all participants (stakeholders and people with intellectual disabilities) at the end of the Stage 1 and Stage 2 focus groups and following the Stage 3 workshop. Items in these questionnaires were taken from Frankena et al.'s (2019) consensus statement on how to conduct inclusive health research, and included items reflecting of personal, professional, research, healthcare, and societal areas. Some longer items were shortened or simplified before being included in the feedback questionnaire. Questionnaires were designed to be quick and simple to complete, and responses were anonymous. Stakeholder participants were asked to endorse any of the 32 items presented to them that reflected their experience of the focus group or workshop. An adapted version was provided for people with intellectual disabilities with 16 items being provided in large text using simple language and with an accompanying image (taken from Easy on the I image bank). Each item was kept to a short sentence (maximum seven words). Respondents could mark yes or no to each item. Yes and no responses were also represented through symbols. Both versions of the questionnaires are available from the authors on request.

Questionnaires were administered by giving a hard copy of the relevant questionnaire to the participant at the end of the focus group or workshop, and ensuring they had a pen or pencil to complete the questionnaire. The response format was explained to the participant, and they were asked if they had any questions. A member of the research team was available in the room throughout the completion of the questionnaire to answer any questions or clarify any items if necessary.

Semi‐structured interviews were conducted, transcribed and anonymised by research assistants from outside of the project to encourage honest feedback on the process. Semi‐structured interview schedules were devised by the research team (based on Fylan et al. 2021) and focused on collecting information about the participants experience of each phase of the project they were involved with, specific processes and techniques within EBCD (such as watching the trigger film, and categorising touchpoints), and interpersonal aspects such as their ability to share thoughts and ideas with other participants. Interviews were conducted with six stakeholders (EBCD01‐06) and eight people with intellectual disabilities (EBCD07‐EBCD13). They were analyzed using Template Analysis by OH. The template was based on Mulvale et al. (2019) and included additional items from Frankena et al. (2019) as well as items arising from the transcripts. Template analysis was chosen as it is a pragmatic and flexible tool allowing particular attention to be paid to material corresponding to the existing consensus statement (which we expected would arise through the interviews), but also allowing flexibility for novel and unexpected areas to be identified.

## 3 Results

Having described the process of adapting EBCD to develop an intervention with people with intellectual disabilities and stakeholders, the amendments to the intervention that came from this process will be detailed. Subsequently, the experiences of participating in the EBCD process will be explored.

### 3.1 What Were the Intervention Refinements Produced Through EBCD and How Were These Implemented?

A total of 24 discreet refinements were described through the EBCD process (see Table 4).

**TABLE 4:** Changes made to the intervention following the EBCD process.

	What we have decided should happen	Why did we decide this?	Who will do it?	By when?	Any barriers that might get in the way?	What will be different when it has been done?
1.	Have a range of prompts to help people develop their mental imagery. These could include physical pictures people could choose from to help them develop their imagery.	To be more person centred	Therapist to discuss with client and supporter in sessions 3 and 4. Could be a homework task after session 3?	OH to add prompt to session materials before study opens for recruitment	Client may not engage with homework task so therapist may need to have some back up options	
2.	Have a recording of the therapist talking through the calm place/ kind helper exercises that the person can use to practice these in between sessions.	To help the client practice new skills and reduce anxiety	Therapist to make recording with client in session 5	Session 5	Client may not have access to device on which to play recording of these exercises. Alternative could be to access online versions that already exist or to use supporter's device.	Client has independent access to accessible materials to practice positive imagery
3.	To check with the client around their preferred terms for the positive image exercises. Might rename the calm place as the ‘happy place’ and the kind helper as a ‘kind person’.	To be more accessible and person centred	Client and therapist in session 5. OH to add prompt to therapist manual to check for this	Session 5		More personalised materials
4.	Change visual analogue scale (VAS) to have 3 or 5 options and use faces to indicate how someone is feeling (used in attention switching task)	To be more personalised and accessible	To have different VAS options available for use in session 6	Session 6		More personalised materials
5.	Use sensory cues such as smells or pictures to help people practice using the calm place and kind helper exercises in between sessions.	To help the client practice new skills and reduce anxiety	Client and therapist (and supporter) to discuss in session 4 or 5. OH to add prompt to therapist manual to check for this and to client workbook	Session 4		Help client remember to practice new skills between sessions
6.	Have discussion with client about the role of supporter at beginning of therapy but make time to revisit this throughout therapy sessions. Also revisit how involved the supporter is and if this needs to be changed.	Need to be flexible to make sure the person is appropriately supported	OH to add written easy read information about supporter and their role to client workbook. OH to add prompt to each therapy session to check with client that they are happy with their supporter and how involved this person is.	Before opening recruitment	None	Written record of role of supporter and what client wants from them
7.	To add information about what a good and bad supporter looks like to the client workbook. To include information about what the person can do if they are not happy with their supporter. Therapist to go through this information with them at beginning of therapy.	To empower clients to think about what they want from their supporter	OH to add written information to client workbook	Before opening recruitment	None	Written record of role of supporter and what client wants from them
8.	Supporter can be involved with planning and preparing around sessions with client	Help client feel more comfortable with session content	OH to add written information to client workbook for client and supporter to suggest this	Throughout intervention	Supporter or client may not want to do this	Better support for client throughout intervention
9.	Reasonable adjustments. Therapist and supporter to check with client throughout the intervention what reasonable adjustments might be helpful and work together with client to implement these	Make intervention more accessible	OH to add prompt for therapist to check accessibility and reasonable adjustments in each therapy session	Throughout intervention	Client may not know what adjustments are needed or be worried about bringing up difficulties in comprehension	Improved accessability
10.	A brief (1 session) component to look at understanding anxiety to be included in the intervention.	More detailed information was not thought to be helpful or necessary	OH	Before opening recruitment to study	None	Basic, written information about anxiety provided
11.	Materials about anxiety should be easy read and have pictures. Only simple text. To be included in a client workbook	Client can look at materials in advance and share them with supporter or staff	OH	Before opening recruitment to study	Literacy skills	
12.	Personalise the materials (e.g., only include information about medication if it is particularly relevant for the person) Have a choice of formats (paper‐based but also electronic options as well)	To be more person‐centred	Therapist/supporter/client throughout intervention	Before opening recruitment to study	Need to check with person and possibly supporter to make sure this is appropriate. May need to be changed or adapted over time	More personalised/ person centred materials
13.	Provide written information about anxiety at screening stage	People would like the information about anxiety before they start the intervention and to be able to go through it with someone they know and trust like a support worker or family member.	Research team	Before opening recruitment to study	None	
14.	Include some information about strategies to help with relaxation at the same time as giving information about anxiety.	People said this would be helpful for them to manage their anxiety	OH	Before opening recruitment to study	None	
15.	At the start of therapy the therapist and client (and maybe supporter) need to have a conversation to make sure the intervention is going to be as accessible as possible. This involves asking the client (and maybe their supporter) to discuss the following with the therapist: How long are sessions? How often are sessions? Where should they take place? Do they prefer a morning or afternoon session? Do they need a break in the session and how could they make this known? What is the persons level of literacy? How do they prefer to communicate (pictures/writing/easy read)? Who else needs information to be shared with them? (family? Supporter? keyworker?) Would they prefer individual or group intervention? Do they need a supporter?	Need to make the intervention person‐centred.	OH to add section to screening session or first therapy session.	Before starting to recruit to intervention	Communication from the service user on what they would like	There will be a document to capture how best to communicate with the client and this can be referred back to in the therapy. This document can be part of the client's workbook.
16.	Add to session 1—as well as discussing confidentiality and the limits of this, discuss the role of supporter and how much information to share with them and how to do this i.e., with client or not, and so forth. Revisit this throughout the therapy (i.e., add as a session point to each therapy session.	Need to have a clear discussion about boundaries, confidentiality and what information to share. This is especially important in a group setting, but also to think about what to share with support of other people	OH to add section to screening session or first therapy session. OH to add prompt to revisit this at end of every therapy session	Before starting to recruit to intervention	None	Written prompt to discuss boundaries in the intervention to remind therapists
17.	Provide easy read summary of each session for client in advance. Could be a one side A4 sheet. Could compile these into a workbook for the intervention for the client so they can share materials with others. Some clients might find it overwhelming to have too much information at once so we could add to the workbook week by week if they prefer that.	So people know what is coming up in sessions. And so they can share their therapy with others if they want. And to remind them of what they have already covered in sessions. Allows for reinforcement of what has been done. Managing expectations. Helps with practicing	OH to make these materials	Before starting to recruit to study	None	Accessible materials provided to client at start of therapy. Resource to refer back to on what each session is and why
18.	Add multi‐sensory items to the therapy if the client enjoys these. They might include music/ sensory prompts etc. to help enhance mental imagery and to increase engagement. Add prompt to session 4 to explore these ideas with clients and revisit in sessions 5, 6 and 7. To include in blueprint if useful for client.	To enhance the vividness of mental imagery and make the therapy more interesting and engaging. Also can use these as cues to practice skills between sessions.	OH to add prompts to discuss in sessions 4,5,6 and 7 and to add to blueprint if appropriate	Before starting to recruit to study	If sensory items cannot be identified. If service user is not calm before explaining plan. If you do not know enough yet	Multi‐sensory prompts added to mental imagery intervention sessions Therapy plan Goal setting
19.	Add relaxation exercise at start of each session	To help client relax and feel calm enough to engage with therapy	OH to add a check in at start of each therapy session with option of doing relaxation exercise/breathing	Before starting to recruit to study	None	Clients offered option of relaxation/breathing to start each session
20.	Offer flexibility around standardised questionnaires—make sure they are as accessible as possible (easy read/pictures/large text). Offer breaks when completing them. Could also complete them with supporter if too many to do all at once.	Questionnaires can be long and boring	OH and/or supporter	Before starting to recruit to study	None	Questionnaires feel more manageable and can be done at client's own pace
21.	To talk with clients and supporter about how they can make the therapy more fun and engaging. Ideas include: Add in pictures/colours the client likes What rewards could they have to help keep them motivated? Certificates at end of therapy? Ending with a game	Therapy needs to be more fun!	OH, client and supporter to discuss in screening session or first therapy session. OH to add section to screening session or first therapy session to prompt for this	Before starting to recruit to study	None	Therapy will feel more fun and engaging for the client
22.	Spend time at start of therapy getting to know client and what their favourite things are eg animals, hobbies, food and so forth	Therapist needs to build rapport and will give them ideas for positive imagery	Therapist in sessions 1 and 2 (and throughout intervention)	Before starting to recruit to study	None	Build therapeutic relationship
23.	Skills practice/clarification. How will the client know when to use these mental imagery skills? What will help them remember to do this?	Need to make sure service user understands when to use it	Psychologist	Before bulk of therapy/early sessions		
24.	Need flexible options for daily recording. Will need to establish what will work best for each client before starting therapy. Need a worksheet for therapist and client (and supporter?) at screening stage to explore how best to do daily recordings. Questions to ask: Electronic or paper format? Would they prefer to use an app (such as Dailyio)? If so what support do they need to set this up? Would they prefer to use text or email? Do they need support to remember to complete a daily measure? Could this be a text reminder (would they like more than 1 reminder a day?) Do they prefer words/emojis/faces/thumbs up or down/numbers to represent how they are feeling? If using paper format what would this look like? And how would they communicate the recordings to the research team?	Need to be person‐centred	OH to devise worksheet	Before opening recruitment	None	Written document of how best to do daily recordings for this person and why

As well as describing refinements, small groups in Stage 3 provided instructions for implementing these refinements, including a rationale for the amendment, who should make the change and within what time frame this should happen through a detailed decision grid. Any barriers to the change were identified and information provided on what would be different within the intervention once the change had been achieved. Groups completed the task given to them which was designed to facilitate a discussion about how best to resolve the issue being presented. Two skilled facilitators guided the group discussion to ensure that all group members were able to continue and that a range of options were explored. During the group discussion one facilitator made notes regarding the topics and ideas discussed as well as ideas or strategies that were rejected by the group. These were fed back to the research team at the end of the workshop. The use of a structured and detailed decision grid which the group completed together allowed for detail about decision taken to be communicated to the research team and reflect the opinions of all group members, rather than relying on the recall of facilitators.

Proposed changes to the intervention ranged in scale from relatively minor amendments such as checking when people may require a break in a therapy session, to much larger changes, such as devising and including a ‘person's workbook’ which encompasses a range of worksheets and information about the different intervention sessions.

It was reassuring that people with intellectual disabilities, and stakeholders were able to engage with the EBCD process and use the workgroups and decision‐making grid to provide a range of feedback and refinements to the interventions. All three mental imagery components presented were retained within the intervention, with various amendments. Decisions included those at a micro level (concerning fine detail of the intervention) as well as overarching ideas, indicating that people were able to hold both multiple aspects of the intervention in mind and provide concrete and specific feedback as to what improvements could be made at each level. Additionally, the feedback provided was extremely helpful. At times the feedback included ideas that had been generated through discussion within the research team and had already been included in the intervention (which was reassuring, indicating that such amendments were endorsed by other stakeholders and service users). Other suggestions for amendments included novel ideas which were then implemented into the intervention before it was tested out within NHS services.

The recommendations from the Stage 3 workshop were largely implemented by the research team. The treatment protocol, therapist's manual and other study documentation were updated to incorporate these refinements, and other new materials were developed (e.g., the person's workbook). All written materials were then circulated to a group of stakeholders for written feedback and further changes to wording, font size, layout, and so forth made to improve accessibility. Individual and small group discussions took place with people with intellectual disabilities to provide feedback on the result of their input at the workshop, and to gain verbal feedback on these additional materials.

### 3.2 What Were the Experiences of Being Involved (Stakeholders, PWID and Researchers) With EBCD (i.e., Was It Acceptable as a Process)?

#### 3.2.1 Results From Questionnaires

Overall results from questionnaires were generally very positive, and feedback for Stage 3 was almost entirely positive.

People with intellectual disability provided overwhelmingly positive feedback on the questionnaires regarding their experiences of the project. They unanimously endorsed that they had been heard within the research, took an active role in the research and that the research was helping to make services accessible and tell others what was important about their health. They described the project as being fun, an opportunity to learn new skills, and to work with other researchers.

Results from the questionnaires completed by stakeholders indicated that people generally enjoyed and engaged with the process. They found that it was meaningful and personally rewarding, as well as contributing to reducing health inequalities. Questionnaires completed by Stakeholders after Focus group 1 and especially Focus group 2 had a smaller number of respondents and showed mixed experiences. Understandably, stakeholder participants in focus groups (in which no participants with intellectual disability were present) were less likely to endorse that the experience was inclusive and heard the voices of people with intellectual disability. It seems that Stage 3 was viewed most positively by both groups, where the mixed discussion groups allowed the inclusive nature of the project to be clearly demonstrated.

#### 3.2.2 Results From Semi‐Structured Interviews

Template Analysis of the semi‐structured interviews identified a total of 27 subthemes which were synthesised into four overarching themes (see Table 5). The subthemes endorsed by each participant was decided by OH after careful reading of the transcript.

**TABLE 5:** Themes endorsed by participants in semi‐structured interviews.

Participant group	Stakeholder participants						Participants with intellectual disability						
	EBCD01	EBCD02	EBCD03	EBCD04	EBCD05	EBCD06	EBCD07	EBCD08	EBCD09	EBCD10	EBCD11	EBCD12	EBCD13
**Working well together**													
Developing trust	✓	✓	✓								✓		✓
Really being listened to	✓		✓		✓	✓	✓	✓		✓			✓
Finding voice	✓		✓	✓	✓	✓	✓	✓			✓	✓	
Having a skilful facilitator	✓		✓		✓	✓	✓			✓		✓	
Ensuring the right mix of participants	✓	✓	✓	✓	✓	✓			✓			✓	
Working together	✓	✓	✓	✓	✓	✓	✓	✓	✓	✓	✓		✓
Enjoying the research process	✓		✓			✓	✓	✓		✓			✓
Good communication	✓	✓	✓				✓						
**Developing research relationships**													
Building community	✓	✓	✓	✓	✓	✓	✓	✓	✓			✓	✓
Feeling connected	✓	✓	✓	✓	✓	✓						✓	✓
Collaboration	✓	✓	✓	✓		✓	✓					✓	✓
Sustaining engagement	✓	✓				✓				✓			
Creating a collective vision	✓	✓	✓		✓	✓							
**Sharing responsibility for the research**													
Sharing power	✓		✓	✓									
Taking on leadership roles in the project	✓		✓	✓		✓				✓			✓
Feeling responsible for the research	✓			✓			✓						✓
Mutual respect	✓	✓								✓			
Research is intellectually challenging				✓		✓						✓	
Intellectual interest in the project	✓	✓	✓		✓	✓	✓		✓			✓	✓
**Making a meaningful contribution to the research**													
Feeling input is valued	✓	✓	✓	✓	✓	✓			✓				
Contributing to the research	✓	✓	✓	✓	✓	✓	✓	✓	✓	✓	✓	✓	
Layering ideas	✓		✓										
Sharing ideas	✓	✓	✓	✓	✓	✓	✓	✓		✓	✓	✓	✓
Sharing different perspectives	✓	✓	✓	✓	✓	✓		✓			✓		
Being able to give honest feedback	✓	✓	✓	✓	✓	✓	✓	✓				✓	
Valuing the ethos of codesign	✓	✓	✓		✓	✓	✓	✓	✓	✓		✓	✓
Meetings need to be accessible						✓	✓		✓	✓	✓		✓

##### 3.2.2.1 Theme 1: Working Well Together

This theme encompassed a number of factors that participants identified as being necessary for the group to work well together. It included each person feeling safe to speak up, and the role of a skilled facilitator in managing discussions. Most participants described having a range of people from different backgrounds and perspectives was helpful to facilitate meaningful discussions. For one participant, the role of the facilitator was key in helping to develop a sense of trust for other participants:

EBCD02: ‘[It] is about the person whose pulling this all together and creating that sort of feeling of collaboration and and giving people a sense of trust that you know getting involved with this will be the safe space’.

Another participant described how, when this skilled facilitation was lacking in an online group, the process felt less safe: ‘And it would have been better if somebody had facilitated that bit because people were just moving these post‐it notes around the screen and they were obviously thinking on a faster level than me. And I was just thinking, “Oh my”’. (EBCD06).

##### 3.2.2.2 Theme 2: Developing Research Relationships

The second theme described various aspects of building and developing a research community. This included sustaining engagement with participants over the 12 months of the EBCD phase. Over this time, people talked about a deepening of connection and relationship with other co‐researchers, which was very much valued, and allowed a collective vision of the research project to crystalise, as this stakeholder described:

EBCD01: ‘When you're in the meetings, you're listening to other people and their ideas, and even if their idea might be completely different to yours or something you've never heard of before. Nobody kind of interrupted each other or anything else […] so everybody got to say what they wanted to, and sometimes we might all agree with stuff. Sometimes we wouldn't, but we would come to because there was something on here about bringing everything together. In the collective voice’.

This process was described by a person with intellectual disabilities:

EBCD10: ‘Yes, I listen to them first and then they listen to me and [we] agreed on things’.

##### 3.2.2.3 Theme 3: Sharing Responsibility for the Research

Within this theme, aspects of sharing responsibility for the research were explored. Interestingly several people (two stakeholders and two people with intellectual disabilities) described the weight of responsibility they felt to get things right for the research project, and to make sure they were putting in sufficient work and effort into the process. This was interesting for the research team to hear and prompted reflections on the potential burdens of involvement in the project. However, when considered alongside the endorsement by six participants of the subtheme ‘taking on a leadership role in the research’, it appeared that people felt able to take an active co‐research role in the project, which is a key aim of EBCD. Therefore, it may be that this feeling of responsibility is a necessary aspect of true co‐design, and thus reflects the level of engagement and investment people felt towards the project. One person with intellectual disabilities described her feelings about the workshop event:

EBCD13: ‘It was a good day. It was a really good day… Seeing lots of people. We were responsible for it in fact’.

##### 3.2.2.4 Theme 4: Making a Meaningful Contribution to the Research

This fourth theme described how people felt their input was meaningful and valuable to the research. As part of this, eight people emphasised the need to give honest feedback to the research team, and to feel comfortable doing this. People described their input into concrete tasks such as producing the trigger films are being valued:

Interviewer: ‘How did you feel about your video being put on that presentation?’

EBCD10: ‘I was happy. They want to know what we're saying and that helped them’.

However, there were occasions when the input from people could not be acted on, and this led to some difficulties for one person:

EBCD12: ‘[researcher] asked us what we would like to do at the workshop and some of it we didn't do because we didn't play bingo and we didn't have music’.

Interviewer: ‘How did that make you feel?’

EBCD12: ‘Upset’.

Overall, there was a strong sense of support for the underlying principles of co‐design, and a sense that having people with intellectual disabilities actively involved in the research was a valuable personal experience and made a genuine difference to the intervention.

EBCD08: ‘To open up. Umm yeah, it's good and. And get our feelings across. Because, like, we need more people with like learning disabilities. Like all all of like people from from all over, like, from all over the country’.

## 4 Discussion

### 4.1 How EBCD Was Adapted to Co‐Design an Intervention for People With Intellectual Disabilities and Stakeholders

Various adaptations were made to the EBCD process, including generic adaptations to make information more accessible for people with intellectual disabilities. Additionally, each stage of the EBCD process was also considered and adapted to enhance engagement. While many adaptations were required, the underlying structure of EBCD remained intact. Both people with intellectual disabilities and stakeholders could engage with the process of EBCD to develop and refine a psychological intervention. They made meaningful and useful contributions to the intervention, including providing a range of concrete and specific ideas for developing and improving the intervention. Generally, participants found the process to be accessible and enjoyed taking part in it. They were able to provide a range of feedback about their experiences, and as such provide valuable insights for others who may wish to use this flexible approach to intervention development.

### 4.2 Refinements Produced Through the EBCD Process

Given that the process of EBCD incorporates multiple, iterative stages, it may have been expected that most of the changes or adaptations the intervention required would have been made and implemented before the final Stage 3 workshop. However, the workshop produced a large number of detailed suggestions for intervention refinement. These focused on both the micro level and broader, overarching principles of the intervention. The use of the decision grid template ensured that feedback was specific and actionable which was helpful to ensure changes could be implemented accurately. The research team were somewhat surprised by the volume of amendments, and as some suggestions required large, new materials to be produced, a considerable amount of work was required to implement these changes in a timely way before the intervention began testing in an NHS setting.

One issue that arose in this project and is described by Fylan et al. (2021), is that while EBCD allows participants to be creative in the solutions they suggest, the research process requires that these solutions are screened and validated, meaning not all suggestions can be adopted. Similarly, one participant was upset that their suggestions for specific food items and the incorporation of games were not able to be included (although several of their other suggestions were incorporated).

### 4.3 Understanding the Experiences of Participating in EBCD

The response rates were very variable for the feedback forms, with high return rates for in person meetings, and very low rates when meetings were held online (i.e., Stakeholder Stage 2), possible due to difficulties in completing online forms. Online sessions were experienced as having pros and cons for stakeholders. They found that skilled facilitation of group work was less successful in online platforms, although the lack of travel meant attending such meetings was easier to manage with competing work demands.

Relatively few men took part in the project. This may be due to the overrepresentation of women in caring and support worker roles, reflecting the persistent cultural norm that care work should be undertaken by women. Similarly, the lack of men with intellectual disability taking part in focus groups or other discussions may be due to a discomfort in discussing mental health issues including anxiety.

Questionnaire feedback was most positive for Stage 3 which may reflect the increased scope of the workshop (e.g., this larger event provided more networking opportunities), as well as understanding how different stages of the process cumulated in the workshop event. This sense of information flowing from one stage to the next may have helped participants understand the overarching process of EBCD and been reflected in more positive evaluations.

Research assistants who were naive to the project and participants (but who had experience of working with people with intellectual disabilities) were employed to conduct semi‐structured interviews. Despite this, interviews were overwhelmingly positive. This contrasts with some of the quantitative feedback from stakeholders (especially regarding Focus groups 1 and 2) which reflected more ambivalent experiences of the process. However, the small number of respondents providing feedback from the stakeholder Focus group 2 was due to the meeting being held online, and feedback was therefore skewed by one participant who found the meeting to be difficult to engage with. Their experience was explored in an interview and fed through in the qualitative results.

Of the four themes identified in the semi‐structured interviews, the first two themes focused on the interpersonal relationships that were needed for individuals to feel safe to participate meaningfully in the research process, and building the research community required to complete the project. The other themes focused on the individual responsibility a person experienced for ensuring the project was completed in a timely way and to a high standard. This was experienced by stakeholders and people with intellectual disability, reflecting the investment they felt in the project. The final theme was around feeling that their input was valuable and had been valued by others. The role of participants at the centre of the EBCD design allowed them to see clearly how invaluable their contribution was.

### 4.4 Challenges and Merits of Using EBCD

The additional burdens and barriers to using co‐design with people with intellectual disabilities have been well documented (e.g., Bishop et al. 2024; Hewitt, Langdon, Tapp et al. 2023). Within this project, additional adaptations needed to be made to ensure that people were able to access meetings, such as holding these at day centres. The Stage 3 workshop event was noisier and more crowded than would have been preferable, due to several participants not providing an RSVP and arriving unexpectedly on the day.

When engaging participants in the EBCD process, materials are presented to elicit discussion and feedback. When presenting the development of the intervention in this project, three parameters were discussed and agreed by the research team. This meant that there were some constraints regarding the direction of the intervention could be taken through the EBCD process. Although these parameters were kept to a minimum and were felt necessary to ensure that an evidence‐based intervention with clear theory of change could be produced, a criticism of the current study may be that providing examples of just three mental imagery components was overly restrictive. Presenting a wider range of potential components may have resulted in different components being selected for the intervention. While the parameter of making the intervention accessible for people with mild to moderate intellectual disabilities was presented by the researcher team, participants in the EBCD process were keen to think about what aspects could be made accessible to people with more severe intellectual disabilities, and in what ways images such as videos and photos could be incorporated into anxiety interventions for this client group.

EBCD has a relatively rigid structure, may be found to lack flexibility (Green et al. 2020). However, we found the clear delineation of the stages of EBCD to be an advantage when working with people with complex and additional needs. The distinct stages of the methodology allowed researchers and those engaging in EBCD to understand clearly each stage of the project, and how each fed into subsequent phases. Thus, we felt that rather than hindering engagement through a lack of flexibility, this rigidity and structure made the methodology more accessible to people with intellectual disabilities.

Epistemic injustice refers to injustice relating to knowledge. It results in people being systematically discredited or neglected and includes silencing and exclusion. Through using the structured and robust framework of EBCD, we ensured that people with limited communication skills were heard from, and their opinions were able to make real change. Qualitative feedback emphasised the sense from people with intellectual disabilities and stakeholders that everyone was genuinely engaged with the project and felt able to make a meaningful contribution. Eleven of the 13 people interviewed expressed that they valued and endorsed the underlying ethos of co‐design, that of sharing power and responsibility for research with people with intellectual disabilities and other stakeholders, and that taking part in the project was in line with this ethos.

### 4.5 Recommendations for Future Studies

The research team found it helpful to use frameworks or templates within the existing literature to guide parts of the EBCD process. EBCD is flexible and can be easily adapted, and we found that the same was true for how we used different frameworks. Consulting examples of how EBCD has been creatively used in other field such as that of child mental health (e.g., Williamson et al. 2021) was helpful. Receiving expert supervision from those with experience of using EBCD in different patient groups and for other purposes was invaluable and provided confidence to implement and adapt EBCD in creative ways to enhance its accessibility. Making connections with other researchers and clinicians using EBCD was also extremely helpful and allowed for the sharing of ideas and resources. Using specific tools such as GRIPP2 (Staniszewska et al. 2017) ensured phases of the project such as the write up, are conducted in accordance with good practice guidance.

While not all participants will be able to take part in every stage of the EBCD process, we found that it was helpful to have a core group of participants who attended all three stages, as this allowed them to develop a wider perspective and deeper understanding of the process. EBCD is a large and complex process with multiple iterative rounds of feedback and refinements to incorporate. It requires good organisation and a proactive stance. Participants that are involved in only one stage of the project may require additional information and support to ensure they have sufficient context and understanding of the overall project.

### 4.6 Conclusions

The process of using EBCD provides a helpful framework for a complex, collaborative process. EBCD can be helpfully adapted to form part of designing complex interventions, and this process can be accessible and meaningful for people with intellectual disabilities.

## Ethics Statement

A favourable ethical opinion for this phase of the project was provided by the Humanities and Social Sciences Research Ethics Committee, University of Warwick (HSSREC 124/20‐21).

## Consent

All participants in the project provided written, informed consent to participate prior to their inclusion in the project.

## Conflicts of Interest

The authors declare no conflicts of interest.

## Data Availability

The data that support the findings of this study are available on request from the corresponding author. The data are not publicly available due to privacy or ethical restrictions.
